# Erythropoietin improves skeletal muscle microcirculation and tissue bioenergetics in a mouse sepsis model

**DOI:** 10.1186/cc5920

**Published:** 2007-05-18

**Authors:** Raymond Kao, Anargyros Xenocostas, Tao Rui, Pei Yu, Weixiong Huang, James Rose, Claudio M Martin

**Affiliations:** 1 Department of National Defense, Canadian Forces Medical Group, 1745 Alta Vista Drive, Ottawa, Ontario, K1A 0K6, Canada; 2 London Health Sciences Center, Divisions of Critical Care & Hematology; Center for Critical Illness Research; Lawson Health Research Institute; University of Western Ontario, 800 Commissioner's Rd. E., London, Ontario, N6A 5W9, Canada

## Abstract

**Introduction:**

The relationship between oxygen delivery and consumption in sepsis is impaired, suggesting a microcirculatory perfusion defect. Recombinant human erythropoietin (rHuEPO) regulates erythropoiesis and also exerts complex actions promoting the maintenance of homeostasis of the organism under stress. The objective of this study was to test the hypothesis that rHuEPO could improve skeletal muscle capillary perfusion and tissue oxygenation in sepsis.

**Methods:**

Septic mice in three experiments received rHu-EPO 400 U/kg subcutaneously 18 hours after cecal ligation and perforation (CLP). The first experiment measured the acute effects of rHuEPO on hemodynamics, blood counts, and arterial lactate level. The next two sets of experiments used intravital microscopy to observe capillary perfusion and nicotinamide adenine dinucleotide (NADH) fluorescence post-CLP after treatment with rHuEPO every 10 minutes for 40 minutes and at 6 hours. Perfused capillary density during a three-minute observation period and NADH fluorescence were measured.

**Results:**

rHuEPO did not have any effects on blood pressure, lactate level, or blood cell numbers. CLP mice demonstrated a 22% decrease in perfused capillary density compared to the sham group (28.5 versus 36.6 capillaries per millimeter; *p *< 0.001). Treatment of CLP mice with rHuEPO resulted in an immediate and significant increase in perfused capillaries in the CLP group at all time points compared to baseline from 28.5 to 33.6 capillaries per millimeter at 40 minutes; *p *< 0.001. A significant increase in baseline NADH, suggesting tissue hypoxia, was noted in the CLP mice compared to the sham group (48.3 versus 43.9 fluorescence units [FU]; *p *= 0.03) and improved with rHuEPO from 48.3 to 44.4 FU at 40 minutes (*p *= 0.02). Six hours after treatment with rHuEPO, CLP mice demonstrated a higher mean perfused capillary density (39.4 versus 31.7 capillaries per millimeter; *p *< 0.001) and a lower mean NADH fluorescence as compared to CLP+normal saline mice (49.4 versus 52.7 FU; *p *= 0.03).

**Conclusion:**

rHuEPO produced an immediate increase in capillary perfusion and decrease in NADH fluorescence in skeletal muscle. Thus, it appears that rHuEPO improves tissue bioenergetics, which is sustained for at least six hours in this murine sepsis model.

## Introduction

Sepsis is a systemic inflammatory response to bacterial infection and is a common complication during the course of treatment of patients in the intensive care unit [1]. On a macroscopic level, significant hematological, hemodynamic, and constitutional instability occurs secondary to the systemic inflammatory response of sepsis. On a microscopic level, there is impairment in the relationship between oxygen delivery (DO_2_) and consumption related to defects in microcirculatory perfusion and disturbances in cellular metabolic pathways, resulting in a deficit of oxygen extraction and use [2-4]. Whether the tissue distress seen in sepsis is caused by microcirculatory hypoxia or disturbances in cellular metabolic pathways is a source of much debate. The debate has been fueled by the findings that despite apparent sufficient DO_2_, signs of cellular hypoxia and metabolic dysfunction persist [5]. Persistent regional tissue dysoxia has been demonstrated in sepsis despite adequate resuscitation procedures that correct global variables of DO_2 _[4]. These observations can be explained, in part, by a pathological redistribution of blood flow giving rise to hypoxic microcirculatory units next to well-perfused or even overperfused normoxic units [6-8]. Even in the absence of systemic hypotension, blood flow and capillary perfusion distribution in both endotoxin and focal models of sepsis can be highly heterogeneous between and within organ systems such as skeletal muscle and the small bowel mucosa [7,9-14].

Studies in critical care support a reduction in the red blood cell (RBC) transfusion threshold [15] and the use of recombinant human erythropoietin (rHuEPO) treatment to reduce transfusion requirements [16-18]. However, besides the regulation of erythropoiesis [19], recent studies indicate that this hormone exerts complex actions promoting the maintenance of homeostasis of the organism under stressors such as oxidation induced during ischemic-reperfusion injury [20-23]. The EPO receptor is distributed in a wide variety of tissues in the cardiovascular system, including cardiomyocytes, vascular smooth muscle, and endothelial cells, and has been shown to mediate anti-apoptotic, anti-inflammatory, and endothelial cell proliferation signaling in a variety of tissue injury models [24]. In addition, rHuEPO may demonstrate vasoactivity that occurs independently of any effects on erythropoeisis and hematocrit. The vasopressor action of rHuEPO may be mediated by several mechanisms, including a direct vasopressor effect on the smooth muscle cells [25,26], and by increasing the circulating plasma levels of the endothelin-1 [27-29]. In a rat splanchnic artery occlusion shock model, treatment with rHuEPO inhibited iNOS (inducible nitric-oxide synthase) activation with restoration of responsiveness to phenylephrine [30,31]. Additionally, rHuEPO may regulate blood flow within the microcirculation through endothelium-dependent mechanisms. Treatment of normal or chronic renal failure patients with rHuEPO induces vasoconstriction of cutaneous capillaries and may improve tissue oxygenation [32,33].

rHuEPO exerts multiple protective actions on the circulatory system, including the microcirculation, which is known to be dysregulated during sepsis. In addition, the blunted endogenous EPO response in critically ill patients with sepsis may contribute further to microcirculatory dysfunction and tissue dysoxia [34]. Based on these observations, we tested the hypothesis that rHuEPO given as a single dose of 400 U/kg would improve skeletal muscle microcirculation and tissue bioenergetics and thus ameliorate tissue metabolic dysfunction in a mouse model of severe sepsis.

## Materials and methods

The University of Western Ontario Council on Animal Care approved the study protocol. Animals were managed according to guidelines set forth by the institutional Council on Animal Care. Mice were acclimatized to the laboratory for one week and had access to mice chow and water *ad libitum*. All surgeries were performed with a clean technique.

### Surgery

The C57BL/6 mice supplied by Charles River Laboratories, Inc. (Wilmington, MA, USA) (ages 10 to 12 weeks; 23 to 26 g) received general anaesthesia with ketamine/xylazine 80:10 mg/kg via intraperitoneal injection. Sepsis was induced by cecal ligation and perforation (CLP). An incision was made along the linea alba. The cecum was mobilized and gently exteriorized using swabs moistened with warm saline (37°C). After ligation, just distal to the ileal cecal valve with 1-0 silk, the cecum was punctured twice with an 18-gauge needle along the anti-mesenteric aspect and gently squeezed to ensure patency of the holes. The cecum was returned to the abdominal cavity and the incision was closed in two layers. In the sham mice group, a similar procedure was performed but without the ligation and puncture. The sham and the CLP animals were allowed to recover with free access to water and mice chow for 18 hours post-surgery prior to treatment with rHuEPO in the CLP group. All animals received buprenorphine 0.1 mg/kg in 1 ml of normal saline (NS) subcutaneously injected after surgery and every eight hours for analgesia and fluid resuscitation. The mice were monitored for signs of discomfort throughout the recovery period.

### Tissue preparation

The sham and the CLP mice were re-anaesthetized and placed on a heating pad, and core temperature was monitored using a thermocouple rectal probe and maintained between 36°C and 37°C. The extensor digitorum longus (EDL) muscle was exposed by gentle dissection. A suture was tied around the distal tendon, which was then separated, and the muscle was reflected over the microscope objective of a Nikon Diaphot 300 inverted microscope (Nikon Canada, Mississauga, ON, Canada) with the proximal neurovascular bundle intact. The preparation then was allowed 45 minutes to equilibrate [35]. To visualize microcirculatory perfusion, the preparation was epi-illuminated with a fiber optic lamp (Schott KL1500; Carl Zeiss Canada Ltd., Toronto, ON, Canada) and images were captured by a video camera (VE-1000CCD; Dage-MTI, Michigan City, IN, USA). Images were displayed on a black and white monitor (WV-BM; Panasonic Corporation of North America, Secaucus, NJ, USA) and were digitally recorded (Liquid Edition version 5.0; Pinnacle Systems, Inc., Mountain View, CA, USA) for analysis [36].

Nicotinamide adenine dinucleotide (NADH) fluorescence from the same area was measured by switching the microscope to an epi-fluorescence configuration using a 100-W mercury arc lamp source, a 365BP25nm excitation filter, a 450BP65 emission filter, and a 400CLP02 dichroic mirror (NADH-specific XF06 filter unit; Omega Optical, Inc., Brattleboro, VT, USA). An additional 550-nm low-pass filter (Omega Optical, Inc.) was installed within the C-mount of the microscope to prevent interference of emission light above 550 nm with the NADH fluorescence image. An ICCD (intensified charge coupled device) camera (IC-110; Photon Technology International, Inc., Birmingham, NJ, USA) captured the images [37].

Capillary density was assessed by observing capillaries of the EDL and counting the number of perfused and stopped capillaries crossing three equidistant lines drawn perpendicular to the direction of the muscle fibers on the observation screen. A capillary was counted as perfused if RBC flow was noted at any time during a three-minute observation period. If there was no flow for the entire three-minute period, the capillary was counted as stopped. Intermittent perfusion was not assessed and plasma-filled (no RBCs visible) capillaries were not detectable with this method. The width of the image field measured was 320 μm per objective field. Capillary density represents the number of capillaries visible across a distance of 1 mm as calculated from the magnification used during the study. NADH fluorescence intensity was measured using Sigma Scan (Jandel Scientific Inc., now part of SPSS Inc., Chicago, IL, USA) and was expressed in arbitrary fluorescence units (FU). To account for fluctuation in daily intensity readings, all data were normalized using a standard NADH solution (41 μmol/l).

### Study protocol

Three sets of experiments were performed. In the first set of experiments, we assessed the acute effects of rHuEPO (Eprex; Ortho Biotech, Toronto, ON, Canada) on hemodynamics, blood cell count, and arterial serum lactate level. In sham and CLP mice at 18 hours after surgery, the mice were re-anesthetized and were given either a 0.2 ml subcutaneous (sc) injection of NS or 400 U/kg rHuEPO (Sham+NS, *n *= 6; Sham+EPO, *n *= 6; CLP+NS, *n *= 7; CLP+rHuEPO, *n *= 7). The 400 U/kg rHuEPO dose was chosen after performing dose-response experiments using single sc doses of 200, 400, 800, and 1,000 U/kg, in which the 400 U/kg dose produced the optimal capillary perfusion during sepsis (data not shown). We measured mean arterial pressure (MAP) by cannulating the carotid artery with Intramedic polyethylene tubing (PE10) (Sparks, MD, USA) connected to a transducer and monitor (78353B; Hewlett-Packard Co., Mississauga, ON, Canada). The heart rate (HR) was determined from a recording of the arterial pressure trace at time 0 and at 40 minutes post-sc injection. Blood samples were also drawn at 40 minutes post-sc injection of saline or rHuEPO for measurement of hemoglobin (Hb), white blood cell (WBC) count, platelets (PLTS), and arterial serum lactate. The complete blood count was measured on an LH750 Series Beckman Coulter hematology analyzer (Beckman Coulter, Fullerton, CA, USA), and the arterial lactate was measured using a VSI 2300 Stat Plus glucose and lactate analyzer (YSI Incorporated, Yellow Springs, OH, USA).

In the second set of experiments, we wished to determined the acute effects of rHuEPO on the microcirculation and NADH levels in the EDL of CLP mice and using the mice as their own baseline control observation at time 0. We performed baseline intravital microscopy in untreated sham mice (*n *= 7) and CLP mice (*n *= 8) 18 hours after surgery. The CLP animals then received a 400 U/kg bolus of rHuEPO by sc injection. Images of the capillaries and NADH fluorescence were recorded every 10 minutes for 40 minutes for both groups.

In the third set of experiments, we wished to determine whether the effects of rHuEPO observed in the second experiment persisted in treated versus untreated CLP mice. Mice underwent CLP and 18 hours later received 0.2 ml sc injections of saline (CLP+NS, *n *= 10) or rHuEPO 400 U/kg (CLP+rHuEPO, *n *= 14). After an additional six hours, the mice were re-anesthetized and intravital microscopy was performed as described above.

### Statistics

The data are expressed as means ± standard error of the mean. Groups were compared at 18 hours before rHuEPO treatment by means of an unpaired *t *test. Within-group comparisons over time were made using a repeated measures analysis of variance, with post hoc paired *t *tests to detect specific differences. A *p *value of less than 0.05 was considered statistically significant.

## Results

Table 1 shows that the CLP group, when compared to the sham, demonstrated an 81% decrease in the WBC count, a 31% decrease in the PLTS, and a 215% increase in arterial serum lactate. CLP also caused a modest drop in MAP from 86 to 73 mm Hg. All of these changes were statistically significant. Treatment with 400 U/kg rHuEPO, however, did not have any effect on the blood pressure, HR, lactate levels, Hb, WBC count, or PLTS at 40 minutes. The Hb level was similar in all four groups (range, 12.1 to 12.8 g/dl). There was no mortality in any of the three series of mice that are reported in this study.

**Table 1:** Hemodynamics, hematology, and lactate measurements in sham and CLP mice

Group	Number	Mean arterial pressure (mm Hg)		Heart rate (beats per minute)		Hemoglobin^a ^ (g/dl)	White blood cell count^a ^ (10^3^/μl)	Platelets^a ^ (10^3^/μl)	Lactate^a ^ (mmol/l)
						
		0 minutes	40 minutes	0 minutes	40 minutes				
Sham+NS	6	86 (1.9)^b^	87 (2.4)	277 (8)	283 (13)	12.7 (0.5)	6.36 (0.84)	1,356.8 (81.3)	0.90 (0.17)
Sham+EPO	6	90 (4.7)	86 (4.3)	294 (11)	293 (4)	12.8 (0.8)	5.97 (1.26)	1,206.7 (128.6)	0.79 (0.18)
CLP+NS	7	73 (1.8)^c^	68 (2.7)^c^	337 (30)	358 (29)	12.1 (0.6)	1.19 (0.35)^c^	938.8 (82.7)^d^	1.94 (0.37)^d^
CLP+EPO	7	70 (2.8)^c^	71 (3.0)^d^	328 (26)	340 (25)	12.7 (0.6)	1.09 (0.29)^c^	956.1 (55.2)	1.91 (0.26)^c^

^a^Blood sample obtained at 40 minutes; ^b^mean (± standard error); ^c^*p *< 0.01 versus corresponding control groups; ^d^*p *< 0.05 versus corresponding control groups. CLP, cecal ligation and perforation; EPO, erythropoietin; NS, normal saline.

**Figure 1:**
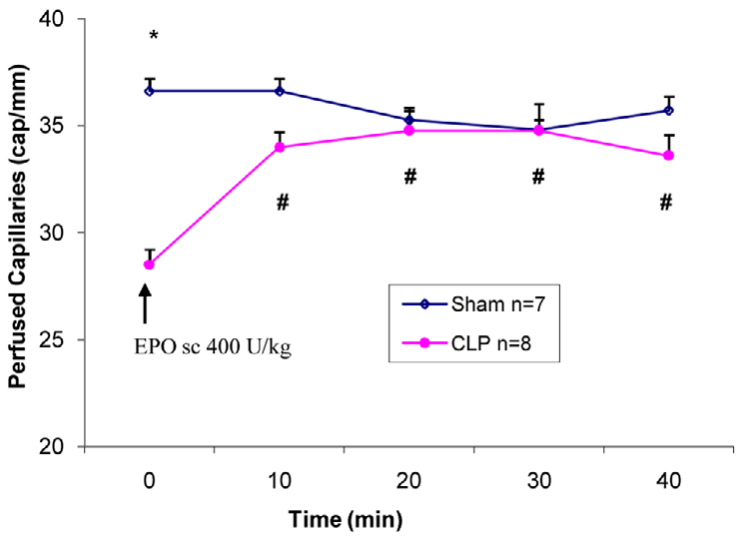
Mean perfused capillary density in sham and cecal ligation and perforation (CLP) mice. The sham and CLP groups consisted of seven and eight mice, respectively. Erythropoietin (EPO) 400 U/kg was administered to the CLP group after baseline measurement (time 0) was obtained.*p < 0.01 versus sham at baseline time 0. #*p *< 0.01 versus CLP at baseline time 0. Values are presented as mean ± standard error. sc, subcutaneous.

**Figure 2:**
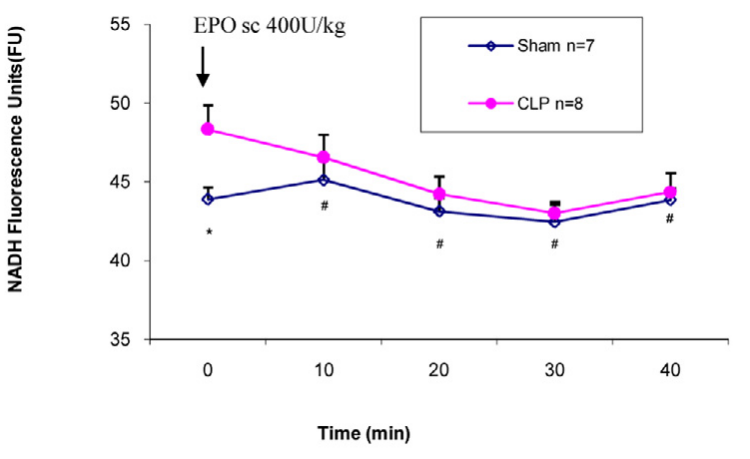
Mean NADH fluorescence unit measurements in sham and cecal ligation and perforation (CLP) mice. The sham and CLP groups consisted of seven and eight mice, respectively. Erythropoietin (EPO) 400 U/kg was administered to the CLP group after baseline measurement (time 0) was obtained. *p < 0.05 versus sham at baseline time 0. #*p *< 0.05 versus CLP at baseline time 0. Values are presented as mean ± standard error. NADH, nicotinamide adenine dinucleotide; sc, subcutaneous.

The baseline comparison at 18 hours after CLP demonstrated an approximately 22% decrease in perfused capillary density in the EDL muscle as compared to the sham group, which was statistically significant (28.5 versus 36.6 capillaries per millimeter; *p *< 0.01; Figure 1). The decreased baseline capillary density was associated with a 10% increase in tissue NADH fluorescence (48.3 versus 43.9 FU; *p *= 0.03; Figure 2). Treatment of the CLP mice with rHuEPO resulted in a significant increase in perfused capillary density to near normal levels by 10 minutes (33.9 versus 28.5 capillaries per millimeter; *p *< 0.001; Figure 1), which persisted until the end of the 40-minute experimental period (33.6 versus 28.5 capillaries per millimeter; *p *< 0.001; Figure 1). Similarly, tissue NADH fluorescence was reduced at 10 minutes (46.5 versus 48.3 FU; *p *= 0.02; Figure 2) and until the end of the 40-minute observation period (44.4 versus 48.3 FU; *p *= 0.02; Figure 2).

**Figure 3:**
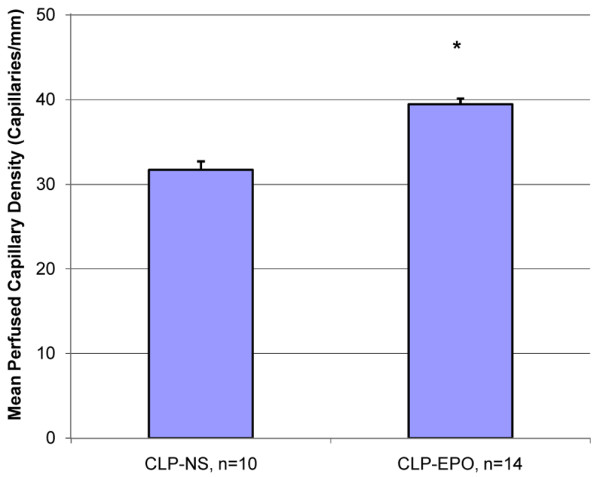
Mean capillary density in cecal ligation and perforation (CLP) mice six hours after treatment with normal saline (NS) or erythropoietin (EPO). Values are presented as mean ± standard error. *p < 0.05 versus CLP-NS.

**Figure 4:**
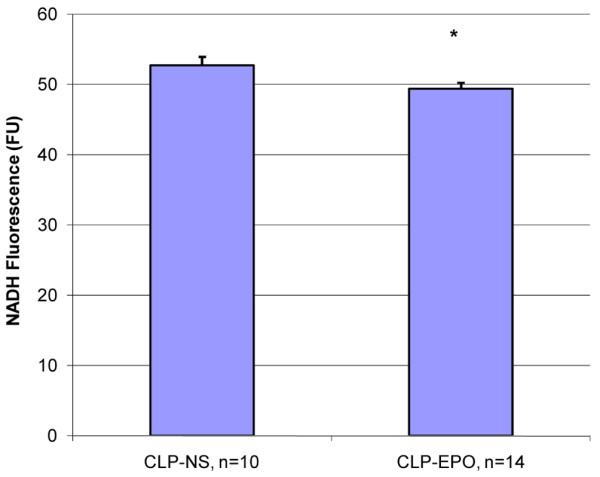
Mean NADH fluorescence units in cecal ligation and perforation (CLP) mice six hours after treatment with normal saline (NS) or erythropoietin (EPO). Values are presented as mean ± standard error. *p < 0.05 versus CLP-NS. NADH, nicotinamide adenine dinucleotide.

In the third set of experiments, CLP mice treated with rHuEPO maintained a significantly higher mean perfused capillary density six hours after rHuEPO injection when compared to CLP mice treated with saline (39.4 versus 31.7 capillaries per millimeter; *p *< 0.001; Figure 3). The increased capillary perfusion was associated with a significantly lower tissue NADH fluorescence at six hours (49.4 versus 52.7 FU; *p *= 0.03; Figure 4).

## Discussion

Many studies in clinical and experimental sepsis have demonstrated that blood flow becomes highly heterogeneous between and within organ systems despite adequate resuscitation [8,35]. This maldistribution of blood flow contributes to abnormal oxygen use at the micro-regional level, leading to tissue injury and organ dysfunction. The CLP mice used in these experiments demonstrated signs of severe sepsis, including a significant drop in blood pressure, an increase in HR, leukopenia, thrombocytopenia, and elevated arterial lactate levels. In this study, we demonstrated that the treatment of septic mice with rHuEPO resulted in increased microcirculatory perfusion, which coincided with a decreased bioenergetic impairment in the skeletal muscle.

The decrease in functional capillary density of approximately 22% induced by sepsis in our mice was similar to the findings of Lam and colleagues [35], in which a 36% reduction in perfused capillary density, a 2.6-fold increase in stopped-flow capillaries, and increased heterogeneity of the spatial distribution of the perfused capillaries were reported in a septic rat EDL model [35]. To determine the functional capillary density, we classified capillaries only as perfused or stopped, with more liberal criteria for perfusion than in the study of Lam and colleagues (any flow over a three-minute period compared to no more than 30 seconds of interrupted flow over a two-minute period). This likely accounts for the relatively decreased effect of sepsis on microcirculation reported in the current study.

The observed loss of functional capillaries appeared to involve individual capillaries as opposed to capillary beds related to single arterioles or venules [35]. After treatment with rHuEPO, the number of perfused capillaries in CLP mice increased within 10 minutes and the improvement in microcirculatory flow persisted for at least six hours. We did not demonstrate an acute effect of rHuEPO on MAP or Hb concentration (Table 1). Thus, it does not appear that direct central vasoactive effects or changes in RBC concentration were factors contributing to the observed improvement of tissue microcirculation.

Coinciding with the increase in the functional capillary density, an improved tissue bioenergetic state was demonstrated using a technique that we have recently validated [36,37]. Treatment with rHuEPO during sepsis resulted in a sustained decrease of tissue NADH fluorescence. An increase in mitochondrial NADH signifies an impairment of electron transport chain (ETC) function. Mitochondrial NADH reduces NADH dehydrogenase (complex I) of the ETC, which further reduces adjacent cytochrome complexes, creating a proton gradient that drives ATP production. Cytochrome C oxidase, the terminal complex of the ETC, reduces molecular oxygen to water, allowing the series of redox reactions of the ETC to continue. If energy transfer ceases anywhere along this pathway, the redox reactions of the ETC will halt, NADH dehydrogenase will remain perpetually reduced, and NADH will accumulate. The accumulation of NADH within mitochondria further affects pyruvate metabolism, which contributes to metabolic acidosis as well. Therefore, we infer that increased NADH fluorescence in the skeletal muscle reflects impaired function of the ETC in these cells and thus a bioenergetic impairment of the tissue.

In the CLP animals, the initial NADH levels at 18 hours were higher than in non-septic controls and normalized following treatment with rHuEPO. The changes in NADH fluorescence were relatively small; however, this observation and the association with a change in microcirculatory perfusion are new observations. At present, we do not have any data that allow us to determine the relation between changes in NADH fluorescence of this magnitude and the prevention of cellular dysfunction or death. Because we did not measure oxidized NADH (NAD^+^), we cannot rule out a change in total pool size contributing to the decrease in NADH fluorescence. Activation of poly (ADP-ribose) polymerase by oxidative stress in sepsis has been proposed as a pathway that could lead to NAD^+ ^depletion, but there is evidence against this occurring in the CLP model [38].

Although we found changes in blood pressure, lactate, WBC count, and PLTS which are consistent with severe sepsis, there was no mortality in this study prior to euthanasia at the completion of each experiment. However, in preliminary studies with this model, we observed a 23% to 25% mortality between 18 to 24 hours post-CLP. This is in contrast to the findings of Hollenberg and coworkers [39], who reported a higher mortality in fluid-treated CLP mice. It is known that mortality in the CLP model is influenced by many factors, including the operator, the laboratory, and groups of mice.

We also recognize that the behavior of the microcirculation in the EDL skeletal muscle during sepsis may not be representative of the microcirculation of other tissues. We chose to use EDL skeletal muscle in this study because it is well characterized in our laboratory and in the literature [35]. Similar changes in the microcirculation of the small bowel mucosa have also been described during sepsis [10,36], but we do not know whether the effects of rHuEPO are generalizable to other tissues.

## Conclusion

rHuEPO treatment in a murine model of severe sepsis induces a rapid normalization in the perfused capillary density with a concomitant decrease in NADH fluorescence in skeletal muscle. Thus, rHuEPO appears to improve mitochondria oxidative phosphorylation and pyruvate metabolism in this septic mouse model in part by improving DO_2 _via increased perfused capillary density. Further studies are warranted to determine the potential mechanisms for these observations and to determine whether this effect is sufficient to improve organ function and reduce morbidity and mortality in sepsis.

## Key messages

• Erythropoietin improves perfused capillary density in the skeletal muscle of septic mice.

• Erythropoietin treatment can also decrease mitiochondrial NADH levels, suggesting improved oxidative phosphorylation and pyruvate metabolism.

• Erythropoietin has a potential clinical application in the improvement of tissue bioenergetics during sepsis.

## Abbreviations

CLP = cecal ligation and perforation; DO_2 _= oxygen delivery; EDL = extensor digitorum longus; ETC = electron transport chain; FU = fluorescence units; Hb = hemoglobin; HR = heart rate; MAP = mean arterial pressure; NAD^+ ^= oxidized nicotinamide adenine dinucleotide; NADH = nicotinamide adenine dinucleotide; NS = normal saline; PLTS = platelets; RBC = red blood cell; rHuEPO = recombinant human erythropoietin; sc = subcutaneous; WBC = white blood cell.

## Competing interests

AX has been an invited speaker and medical monitor for Ortho Biotech and the recipient of unrestricted educational grant from Ortho Biotech. The other authors declare that they have no competing interests.

## Authors' contributions

RK conceived of and designed the study, performed data analysis, drafted the manuscript, and approved the final version of the manuscript. CMM conceived of and designed the study, revised the manuscript for critically important intellectual content, and approved the final version of the manuscript. AX conceived of the study, drafted the manuscript for critically important intellectual content, and approved the final version of the manuscript. TR revised the manuscript for critically important intellectual content and approved the final version of the manuscript. PY, WH, and JR carried out the animal experiments and acquisition of data. RK and AX contributed equally in drafting this manuscript.

## References

[B1] Levy MM, Fink MP, Marshall JC, Abraham E, Angus D, Cook D, Cohen J, Opal SM, Vincent JL, Ramsay G (2003). 2001 SCCM/ESICM/ACCP/ATS/SIS International Sepsis Definitions Conference. Crit Care Med.

[B2] Nelson DP, Samsel RW, Wood LDH, Schumacker PT (1988). Pathological supply dependence of systemic and intestinal O_2 _uptake during endotoxemia. J Appl Physiol.

[B3] Cain SM, Curtis SE (1991). Experimental models of pathologic oxygen supply dependency. Crit Care Med.

[B4] Gattinoni L, Brazzi L, Pelosi P, Latini R, Tognoni G, Pesenti A, Fumagalli R (1995). A trial of goal-oriented hemodynamic therapy in critically ill patients. SVO_2 _Collaborative Group. N Engl J Med.

[B5] Ince C, Sinaasappel M (1999). Microcirculatory oxygenation and shunting in sepsis and shock. Crit Care Med.

[B6] Uusaro A, Ruokonen E, Takala J (1995). Gastric mucosal PH does not reflect changes in splanchnic blood flow after cardiac surgery. Br J Anaesth.

[B7] Madorin WS, Martin CM, Sibbald WJ (1999). Dopexamine attenuates flow motion in ileal mucosal arterioles in normotensive sepsis [see comments]. Crit Care Med.

[B8] Bersten A, Sibbald WJ (1989). Circulatory disturbances in multiple systems organ failure. Crit Care Clin.

[B9] Drazenovic R, Samsel RW, Wlam ME, Doerschuk CM, Schumacker PT (1992). Regulation of perfused capillary density in canine intestinal mucosa during endotoxemia. J Appl Physiol.

[B10] Farquhar I, Martin CM, Lam C, Potter R, Ellis C, Sibbald WJ (1996). Decreased capillary density *in vivo *in bowel mucosa of rats with normotensive sepsis. J Surg Res.

[B11] Hersch M, Madorin S, Martin CM, Sibbald WJ (1998). Selective gut microcirculatory control (SGMC) in septic rats: a novel approach with a locally applied vasoactive drug. Shock.

[B12] Schinke M, Doods GD, Wienen W, Entzeroth M (1991). Characterization of rat intestinal angiotensin II receptors. Eur J Pharmacol.

[B13] Tugtekin IF, Radermacher P, Theisen M, Matejovic M, Stehr A, Ploner F, Matura K, Ince C, Georgieff M, Trager K (2001). Increased ileal-mucosal-arterial PCO_2 _gap is associated with impaired villus microcirculation in endotoxic pigs. Intensive Care Med.

[B14] Whitworth PW, Cryer HM, Garrison RN (1989). Hypoperfusion of the intestinal microcirculation without decreased cardiac output during live *Escherichia coli *sepsis in rats. Circ Shock.

[B15] Hebert PC, Wells G, Blajchman MA, Marshall J, Martin CM, Pagliarello G, Tweeddale M, Schweitzer I, Yisiter E (1999). A multicentre, randomized, controlled clinical trial of transfusion requirements in critical care. N Engl J Med.

[B16] Van Iperen CE, Gaillard CA, Kraaijenhagen RJ, Braam BG, Marx JJ, Vande WA (2000). Response of erythropoiesis and iron metabolism to recombinant human erythropoietin in intensive care unit patients. Crit Care Med.

[B17] Corwin HL, Gettinger A, Rodriguez R, Pearl RG, Gubler KD, Enny C, Colton T, Corwin MJ (1999). Efficacy of recombinant human erythropoietin in the critically ill patient: a randomized, double-blind, placebo-controlled trial. Crit Care Med.

[B18] Gabriel A, Kozek S, Chiari A, Fitzgerald R, Grabner C, Geissler K, Zimpfer M, Stockenhuber F, Bircher NG (1998). High dose recombinant human erythropoietin stimulates reticulocyte production in patients with multiple organ dysfunction syndrome. J Trauma.

[B19] Jelkmann W (1992). Erythropoietin: structure, control of production and function. Physiol Rev.

[B20] Solaroglu I, Solaroglu A, Kaptanoglu E, Dede S, Haberal A, Beskonakli E, Kilinc K (2003). Erythropoietin prevents ischemic-reperfusion from inducing oxidative damage in fetal rat brain. Childs Nerv Syst.

[B21] Bany-Mohammed FM, Slivka S, Hallman M (1996). Recombinant human erythropietin: possible role as an antioxidant in premature rabbits. Pediatr Res.

[B22] Eid T, Brines M (2002). Recombinant human erythropoietin for neuroprotection: what is the evidence?. Clin Breast Cancer.

[B23] Buemi M, Cavallaro E, Floccari F, Sturiale A, Aloisi C, Trimarchi M, Grasso G, Corica F, Frisina N (2002). Erythropoietin and the brain: from neurodevelopment to neuroprotection. Clin Sci (Lond).

[B24] van der Meer P, Voors AA, Lipsic E, van Gilst WH, van Veldhuise DJ (2004). Erythropoietin in cardiovascular diseases. Eur Heart J.

[B25] Heidenreich S, Rahn KH, Zidek W (1991). Direct vasopressor effect of recombinant human erythropoietin on renal resistance vessles. Kidney Int.

[B26] Neusser M, Tepel M, Zidek W (1993). Erythropoietin increases cytosolic free calcium concentration in vascular smooth muscle cells. Cardiovasc Res.

[B27] Takahashi K, Totsune K, Imai Y (1993). Plasma concentration of immunoreactive-endothelin in patients with chronic renal failure treated with recombinant human erythropoietin. Clin Sci.

[B28] Carlini R, Dusso A, Chamberlain L, Obialo C, Alvarez U, Rothstein M (1993). Recombinant human erythropoietin (rHuEPO) increases endothelin-1 release by endothelial cells. Kidney Int.

[B29] Liefeldt L, Schmidt-Ott KM, Orzechowski H-D (1998). Transcriptional regulation of endothelin-1 by erythropoietin in endothelial cells. J Cardiovasc Pharmacol.

[B30] Buemi M, Allergra A, Squadrito F, Uemi AL, Lagana A, Aloisi C, Frisina N (1993). Effects of intravenous administration of recombinant human erythropoietin in rats subjected to hemorrhagic shock. Nephron.

[B31] Squadrito FD, Altavilla G, Squadrito GM, Campo M, Arlotta C, Quartarone A, Siatta A, Caputi AP (1999). Recombinant human erythropoietin inhibits iNOS activity and reverts vascular dysfunction in splanchnic artery occlusion shock. Br J Pharmacol.

[B32] Buemi M, Denuzzo G, Allegra A, Aloisi C, Squadrito G, Dattola A, Corica F, Vermiglio G (1995). Recombinant human erythropoietin inhibits the cutaneous vasodilatation induced by acetylcholine. Int J Microcirc Clin Exp.

[B33] Creutzig A, Caspary L, Nonnast-Daniel B, Bahlmann J, Kuhn K, Brunkhorst R, Reimers E, Koch KM, Alexander K (1990). Skin microcirculation and regional peripheral resistance in patients with chronic renal anaemia treated with recombinant human erythropoietin. Eur J Clin Invest.

[B34] Rogiers P, Zhang H, Leeman M, Nagler J, Neels H, Mélot C, Vincent JL (1997). Erythropoietin response is blunted in critically ill patients. Intensive Care Med.

[B35] Lam C, Tymal K, Martin CM, Sibbald WJ (1994). The skeletal muscle microcirculation in a rat model of normotensive sepsis. J Clin Invest.

[B36] Rose J, Martin CM (2006). Tissue bioenergetics and microvascular perfusion are impaired in rat ileal mucosa in normotensive sepsis. Microcirculation.

[B37] Rose J, Martin CM, MacDonald TL (2006). High-resolution intravital NADH fluorescence microscopy allows measurements of tissue bioenergetics in rat ileal mucosa. Microcirculation.

[B38] Baechtold F, Scott JA, Market M, Mehta S, McCormack DG, Anglada F, Galaud D, Vaglio M, Waeber B, Feihl F (2001). Does the activation of poly (ADP-ribose) synthetase mediate tissue injury in the sepsis induced by cecal ligation and puncture?. Shock.

[B39] Hollenberg SM, Dumasius A, Easington C, Colilla S, Neumann A (2001). Characterization of a hyperdynamic murine model of resuscitated sepsis using echocardiography. Am J Respir Crit Care Med.

